# Synthesis, conformation and Hirshfeld surface analysis of benzoxazole methyl ester as a versatile building block for heterocycles

**DOI:** 10.1016/j.heliyon.2021.e08042

**Published:** 2021-09-21

**Authors:** Aamer Saeed, Ghulam Shabir, Tuncer Hökelek, Ülrich Flörke, Mauricio F. Erben

**Affiliations:** a Department of Chemistry, Quaid-I-Azam University, 45320, Islamabad, Pakistan; b Department of Physics, Faculty of Engineering, Hacettepe University, 06800, Beytepe-Ankara, Turkey; c Department Chemie, Fakultät für Naturwissenschaften, Universität Paderborn, Warburgerstrasse 100, D-33098, Paderborn, Germany; d CEQUINOR (UNLP-CONICET, CCT-La Plata), Departamento de Química, Facultad de Ciencias Exactas, Universidad Nacional de La Plata, Boulevard 120 e/ 60 y 64 Nº 1465 La Plata, B1900, Buenos Aires, Argentina

**Keywords:** Molecular structure, benzo[*d*]oxazole-2(3*H*)-thione, Hirshfeld surface analysis, Natural bond orbital

## Abstract

Solventless cyclocondensation of 2-aminothiophenol with thiourea afforded the benzo[*d*]oxazole-2-thiol (**3a**) capable of existing also in the tautomeric form benzo[*d*]oxazole-2(3*H*)-thione (**3b**). Acylation with methyl chloroacetate in dry ethanol in absence of any base or catalyst selectively afforded the *S*-substituted ester 2-(methoxycarbonylmethylthio)benzo[*d*]oxazole (**4a**) in preference to the corresponding *N*-substituted ester *N*-(methoxycarbonylmethyl)thioxobenzoxazole (**4b**). Quantum chemical calculations were conducted to determine the conformational landscape and NBO population analysis showed the strong electronic delocalization *via* resonance interactions on the 2-mercaptobenzaxazole group. The anomeric effect and the occurrence of a 1,4-S···O intramolecular interactions suggest the relevance of chalcogen bonding in the conformational preference. The Hirshfeld surface analysis of the crystal structure indicates that the most important contributions for the crystal packing are from H⋯H (33.2%), H⋯O/O⋯H (19.9%) and H⋯C/C⋯H (17.8%) interactions. Hydrogen bonding and van der Waals interactions are the dominant interactions in the crystal packing. Computational chemistry indicates that in the crystal, the C–H⋯O hydrogen-bond energy is 44.8 kJ mol^−1^.

## 1 Introduction

Benzoxazole-2-thiol like its nitrogen and sulfur analogues 1*H*-benzoimidazole-2-thiol and benzothiazole-2-thiol respectively, is capable of existing in tautomeric thione-thiol isomerism involving –NH-C=S and –N=C-SH groups in equilibrium. These are ambidentate ligands towards transition metal ions and have an important role in industry and medicine [1]. Only the exocyclic sulfur and the nitrogen atom of the ring can take part in coordination as the lone pairs on the oxygen are involved in the resonance [2].

Although a variety of 1-acylthiosemicarbazides derived from 2-benzoxazolinones and 2-benzothiazolinone and a range of heterocycles: 1,2,4-triazole-5(4*H*)-thiones, 1,3,4-thiadiazo- les, 1,3,4-oxadiazoles and hydrazones obtained by varying the cyclization conditions have been reported, those derived from benzoxazole-2-thione are relatively rare in literature [3]. In particular, 2-mercapto benzoxazoles were used as versatile precursors for the formation of polymer modified electrodes in aqueous solution [4]. Sulfur substituted heterocyclic compounds represent an important group of sulphur compounds with promising practical applications. Among these heterocycles, mercapto- and thione-substituted 1,2,4-triazole ring systems are showing a variety of biological activities ranging from antimicrobial to anticancer properties. 1,3,4-thiadiazoles and their condensed derivatives comprise another class of organic compounds with analgesic-antiinflammatory [5] and antimicrobial activities [6, 7].

Recent efforts in the preparation of substituted-thio-benzo[*d*]oxazoles have been reported [8, 9]. In particular, the title compound was prepared as a key intermediate towards a variety of *S*-substituted heterocycles in order to explore them for comparison of their biological activities with corresponding well-known *N*-substituted analogues [10]. Thus, the acid hydrazide synthesized by the condensation of methyl 2-(benzo[*d*]oxazol-2-ylthio) acetate with hydrazine hydrate could be converted into thiosemicarbazides by the reaction with substituted isothiocyanates [11]. On cyclization, the latter in presence of base should afford 1,2,4-triazoles whereas the acid catalyzed cyclization will afford 1,3,4-thiadiazoles [12, 13]. The treatment with aromatic aldehydes will result in the formation of arylidene hydrazides [14].

In this contribution, improved selective synthesis and spectroscopic characterization of 2-(methoxycarbonylmethylthio)benzo[*d*]oxazole is reported. The conformational landscape is analyzed by using computational methods and the intermolecular interactions are studied by applying the Hirshfeld surface analysis calculations. Moreover, the electronic properties around the central 2-mercaptooxazole group are analyzed by using NBO population analysis.

## 2 Experimental section

Melting points were recorded using a digital Gallenkamp (SANYO) model MPD BM 3.5 apparatus and are uncorrected. ^1^H NMR spectra were determined as CDCl_3_ solutions at 300 MHz using a Bruker AM-300 spectrophotometer. FT IR spectra were recorded using an FTS 3000 MX spectrophotometer, Mass Spectra (EI, 70 eV) on a GC-MS instrument Agilent technology, and elemental analyses were conducted using a LECO-183 CHNS analyzer. Both compounds were recrystallized from aqueous ethanol.

### 2.1 Synthesis of benzo[*d*]oxazole-2-thiol (3)

A stirred, homogenized mixture of 2-aminophenol (1 mmol) (**1**) and thiourea (1 mmol) (**2**) was heated at 200 °C for 2 h. The progress of the reaction was followed by TLC examination using hexane: ethyl acetate (9:1). On completion, the product was recrystallized using ethanol to afford compound (**3**) in 74% yield. M.p and spectroscopic data agreed well to that reported in literature [15].

### 2.2 Synthesis of 2-(methoxycarbonylmethylthio)benzo[*d*]oxazole (4)

Equimolar quantities of benzo[d]oxazole-2-thiol (**1**) and methyl chloroacetate in dry methanol were refluxed for 6 h. On completion as indicated by analytical TLC, the reaction mixture was cooled to room temperature and poured to the crushed ice. The product precipitated out was filtered, washed with water, dried and recrystallized from ethanol to afford (**4**) (1.7 g, 82%). m.p. 146–147 C [10]. IR (KBr cm^−1^): 1735 (C=O), 1228 1131 (C–O). ^1^H NMR: 7.3–7.6 (m, 4H, Ar), 4.1 (s, 2H), 3.6 (s, 3H). ^13^C NMR: 169.5 (C=O), 165.0 (C=N), 150.0, 141.5, 124.9, 123.9, 119.2, 110.7, 51.2, 31.9. GC-EI-MS: m/z: 223, Anal. Calcd for C_10_H_9_NO_3_S: C, 53.80; H, 4.06; N, 6.27; S, 14.36, Found: C, 53.89; H, 4.10; N, 6.22; S, 14.31%.

### 2.3 Computational details

Molecular quantum chemical calculations have been performed with the GAUSSIAN 03 program package [16] by using the B3LYP DFT hybrid methods employing Pople-type basis set [17] for geometry optimization and frequency calculations, as suggested by Saglam et al. for related heterocyclic compounds [18]. The calculated vibrational properties corresponded in all cases to potential energy minima for which no imaginary frequency was found. Natural population analysis and second-order Donor→ acceptor interaction energies were estimated at the B3LYP/6–311++G(d,p) level by using the NBO analysis [19] as implemented in the GAUSSIAN 03 program.

## 3 Results and discussion

### 3.1 Chemistry

**Figure 1:**
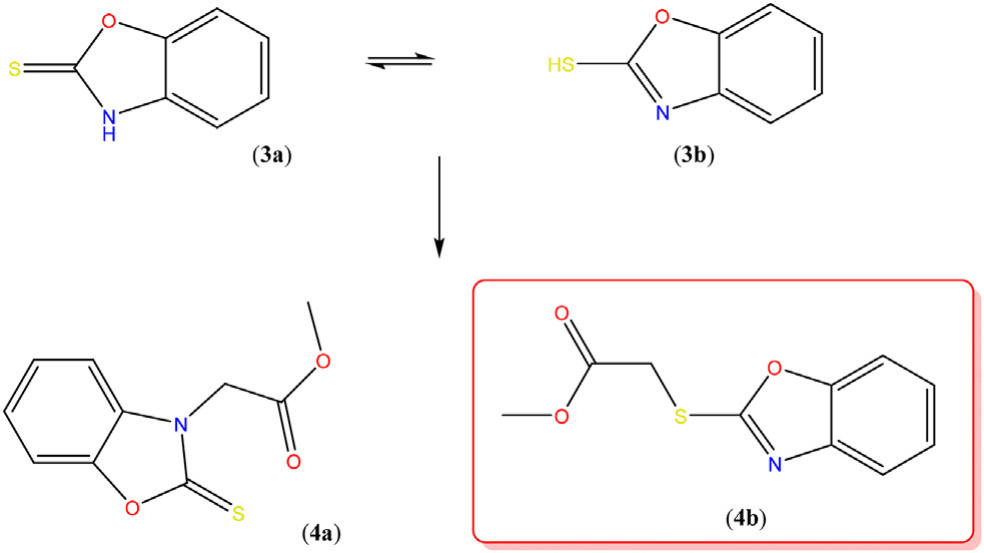
Thione-thiol tautomerism in benzo[*d*]oxazole-2-thiol (**3**) leading to *N*-substituted product (**4a**) or *S*-substituted product (**4b**) upon reaction with methyl 2-chloroacetate.

The cyclocondensation of 2-aminothio phenol (**1**) with thiourea (**2**) afforded compound (**3**) which is capable of existing as thione form benzo[*d*]oxazole-2(3*H*)-thione (**3a**) or thiol tautomer, benzo[*d*]oxazole-2-thiol (**3b**). Therefore on reaction with methyl 2-chloroacetate, it can produce either the *S*-substituted product methyl 2-(2-thioxobenzo[*d*]oxazol-3(2*H*)-yl)acetate (**4a**) or the corresponding *N*-substituted product methyl 2-(benzo[*d*]oxazol-2-ylthio)acetate (**4b**) (Figure 1).

**Figure 2:**
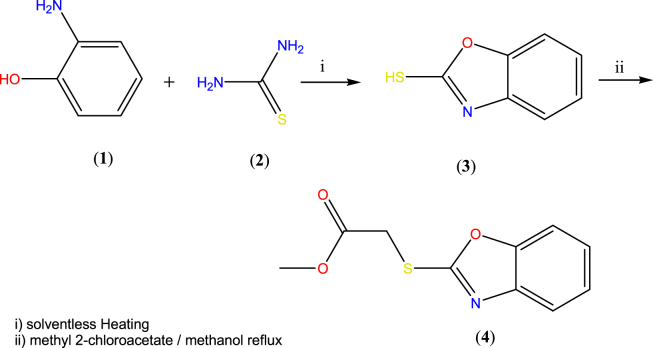
Synthetic pathway to ester of benzoxazole.

Previous reports to the synthesis of (**3**) involve the reaction of 2-aminophenol with potassium hydroxide in liquid carbon disulfide along with long reaction times, and difficult workup [15]. Earlier we had also reported a similar cyclo-condensation reaction promoted by microwave radiation [10], however currently it was found that simple fusion of the two reactants under conventional heating gave the product in better yield. Consequently, by modification of our method [10], solventless synthesis of benzo[*d*]oxazole-2-thiol (**3**) was achieved by direct cyclocondensation of 2-aminothio phenol (**1**) with thiourea (**2**) in absence of solvent or catalyst in good yield and high purity. Similarly, the reaction of equimolar amounts of methyl chloroacetate and benzoxathiole (**3**) could be carried out dry methanol under reflux conditions in the absence of any base to afford ester (**4b**) shown in Figure 2.

Formation of (**4**) from (**3**) was initially indicated by the absence of the N–H/S–H stretching absorptions at *ca*. 3270/2560 cm^−1^ and appearance of characterizing bands of the ester group at 1735 (C=O), 1131 (C–O) in the FTIR spectrum. That the product is (**4b**) and not (**4a**) was indicated by appearance of methylene singlet at δ= 4.1 ppm in the ^1^H NMR spectrum, which should be slightly more deshielded when flanked by carbonyl and nitrogen. In the ^13^C NMR spectrum, the peaks δ= 169.5 ppm (C=O) and 165.0 ppm (C=N) and the absence of thiocarbonyl peak was noted.

### 3.2 Conformational study and natural bond population analysis (NBO)

**Figure 3:**
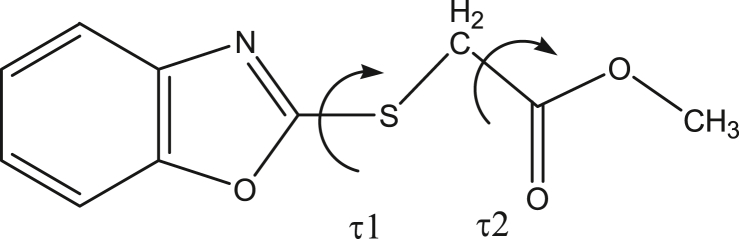
Definition of the τ1 and τ2 dihedral angle for the 2-(carbonylmethylthio) oxazole moiety.

The 2-(carbonylmethylthio) oxazole moiety is very interesting from the conformational point of view, since internal rotation around two dihedral angles, i.e. C7–S1 and C8–C9 (see Figure 3), gives rise to different conformations.

Thus, following Zeyrek et al. [20], the conformational landscape was firstly analyzed by performing quantum chemical calculations to know the potential energy function for internal rotation around each of the dihedral angles at the B3LYP/6-31G(d) level of approximation. Each minima was further submitted to geometry re-optimization and frequency calculations with the more extended valence triple-ξ basis set augmented with diffuse and polarization functions in both the hydrogen and weighty atoms [6–311++G(d,p)]. The calculated vibrational properties corresponded in all cases to potential energy minima for which no imaginary frequency was found.

**Figure 4:**
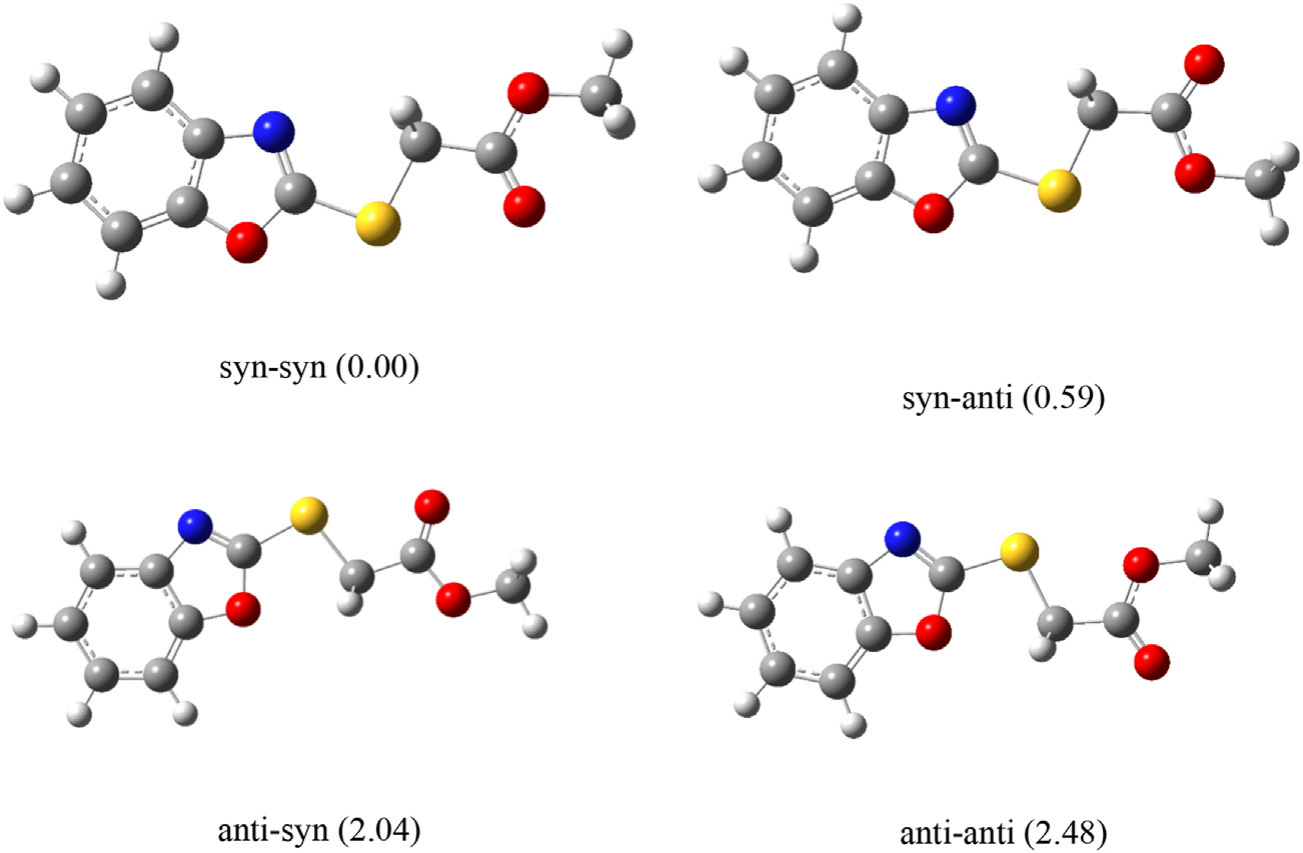
Four most stable conformers optimized at the B3LYP/6–311++G(d,p) level of approximation for the title compound, with relative electronic energy in kcal/mol.

Two minima are found for rotation of τ1, these are planar conformers with the S–C single bond oriented toward the C=N or the C–O bonds in the oxazole ring. The most stable form presents a synperiplanar orientation between the C=N double bond and the S–C single bond. The whole molecule is planar, belonging to the *C*_S_ punctual symmetry group (it is identified as the syn-syn form in Figure 4). This syn-syn conformation coincides with the experimental molecular structure found in the crystal [10], where the five- and six-membered rings of the benzo[d]oxazole moiety are almost coplanar.

The second conformation, with the S–C bond oriented toward the oxygen atom in the oxazole ring is higher in energy by 2.04 kcal/mol, identified as anti-syn (Figure 4).

When τ2 is analyzed, also two planar conformations are found to be minima in the potential energy curve, depending on the mutual orientation of the C=O double bond and the C–S single bond. The most stable conformation shows the synperiplanar orientation, with the antiperiplanar one being higher in energy by only 0.59 kcal/mol (anti-syn in Figure 4). It is worthy to note that both conformations promote close 1,4-sulfur-oxygen interactions, between the sulfur and oxygen atoms from the carbonyl or methoxy groups, respectively.

The computed electronic energy of the least stable anti-anti conformer is 2.48 kcal/mol, as shown in Figure 4.

The planar forms adopted by the four studied conformations suggest the occurrence of strong electronic delocalization promoted by π resonance across the entire molecule. To better understand this effect, electronic interactions have been analyzed with the NBO population analysis [21]. The main second-order perturbation stabilization energies, E^(2)^, computed (B3LYP/6–311++G(d,p)) for the four conformations are given in Table 1.

**Table 1:** Selected second order donor → acceptor interaction energies (E^(2)^ in kcal/mol) computed (B3LYP/6–311++G(d,p)) for the four conformers of the studied compounds.

Donor →acceptor interaction	syn-syn	syn-anti	anti-syn	anti-anti
lpσ(S1)→ σ∗(C7 = N1)	6.51	6.43	-	-
lpσ(S1)→ σ∗(C7–O1)	-	-	5.34	5.29
lpπ(S1)→ π∗(C7 = N1)	29.09	29.11	27.73	27.39
lpσ(O1)→ σ∗(C7 = N1)	4.75	4.77	4.38	4.40
lpπ(O1)→ π∗(C7 = N1)	33.84	34.05	32.47	32.70
lp(N1)→ σ∗(C7–O1)	12.80	13.23	14.05	14.00
lpπ(O2)→ π∗(C9 = O3)	47.86	48.35	47.72	48.91
lpπ(O1)→ π∗(C=C)	22.05	21.96	21.27	21.27
lp(O3)→ σ∗(S1–C7)	0.89	-	-	-

The results for the syn-syn conformer indicate the presence of p-type out-of-plane lone pair orbitals at sulfur and oxygen atoms, in which the electron-donating capacity is reflected by its low electron occupancy, of *ca*. 1.861, 1.744, 1.792 and 1.845 **e**, for S1, O1, O2 and O3, respectively. The interactions in the oxazole ring include lp(N1)→ σ∗(C7–O1), lpπ(O2)→ π∗(C7 = N1), with 12.80 and 33.84 kcal/mol, respectively, and conjugations with the benzo ring through the lpπ(O2)→ π∗(C=C) resonance. The 2-thio substitution favors a strong resonance between the sulfur atom and the oxazole group, with the lpπ(S1)→ π∗(C7 = N1) interaction amounting up to 29.09 kcal/mol for the most stable conformer. Similar resonance interaction energies were reported for related heterocycles [22, 23]. Furthermore, the π-electron system is also extended over the methoxycarbonyl group, with strong lpπ(O2)→ π∗(C=O3) of *ca*. 48 kcal/mol.

Since the directional nature of the NBOs, hyperconjugative interactions depend on the specific conformation. The in-plane lpσ(S1) lone pair located at the sulfur atom overlaps with the σ∗(C7 = N1) for the syn conformation of the C–S and C–N bond, with E^(2)^ energy of ca. 6.5 kcal/mol. On the other hand, overlapping between lpσ(S1) and C7–O1 via the lpσ(S1)→ σ∗(C7–O1) donor-acceptor interaction is favored for the anti conformation, with E^(2)^ values of *ca*. 5.3 kcal/mol. Thus, the anomeric effect seems to play a key role in the conformational preference around the 2-thio oxazole moiety. Similar contributions of the lpσ(S1) was early reported for the case of methoxycarbonyl sulfenyl compounds [24].

Finally, it is worthy to indicate the occurrence of a remote 1,4-interaction [25] between electron density of the oxygen atom of the carbonyl group (C9 = O3) and the σ∗(S1–C7) antibonding orbital. This lp(O3)→ σ∗(S1–C7) is weak (0.89 kcal/mol) but it is only observed for the most stable syn-syn conformation. This chalcogen···chalcogen interaction favors the occurrence of a pseudo four-membered ring, with S1⋯O4 distance of 2.781(4)Å in the crystal [10].

### 3.3 Hirshfeld surface analysis

**Figure 5:**
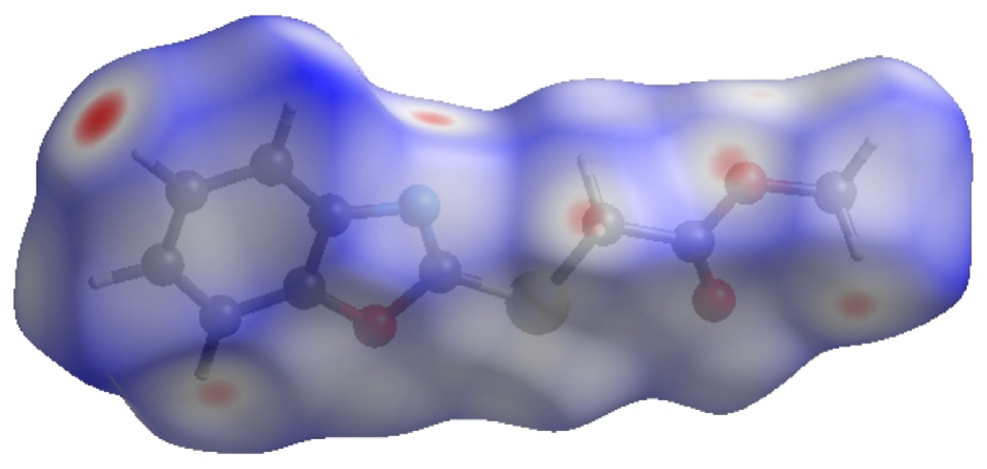
View of the three-dimensional Hirshfeld surface of the title compound plotted over d_norm_ in the range of -0.2272 to 1.2126 a.u.

**Figure 6:**
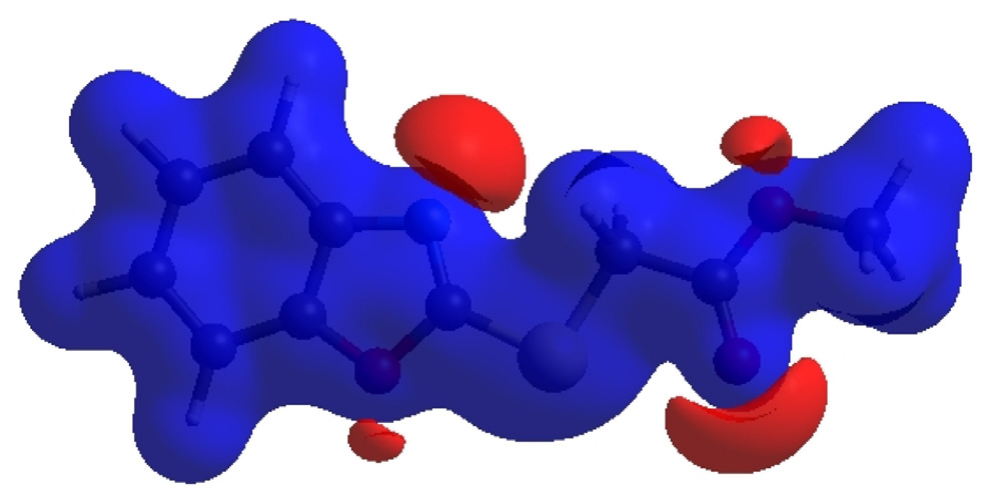
View of the three-dimensional Hirshfeld surface of the title compound plotted over electrostatic potential energy in the range -0.0500 to 0.0500 a.u. Hydrogen-bond donors and acceptors are shown as blue and red regions around the atoms corresponding to positive and negative potentials, respectively.

**Figure 7:**
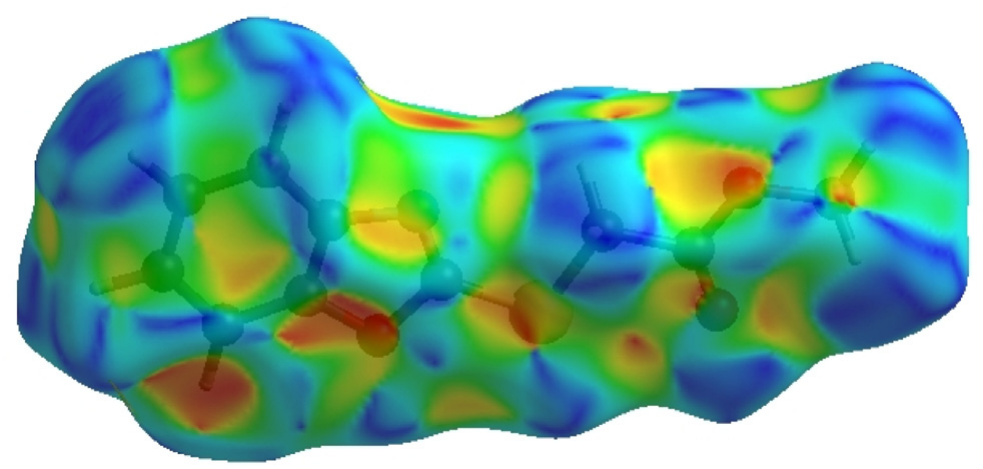
Hirshfeld surface of the title compound plotted over shape-index.

**Figure 8:**
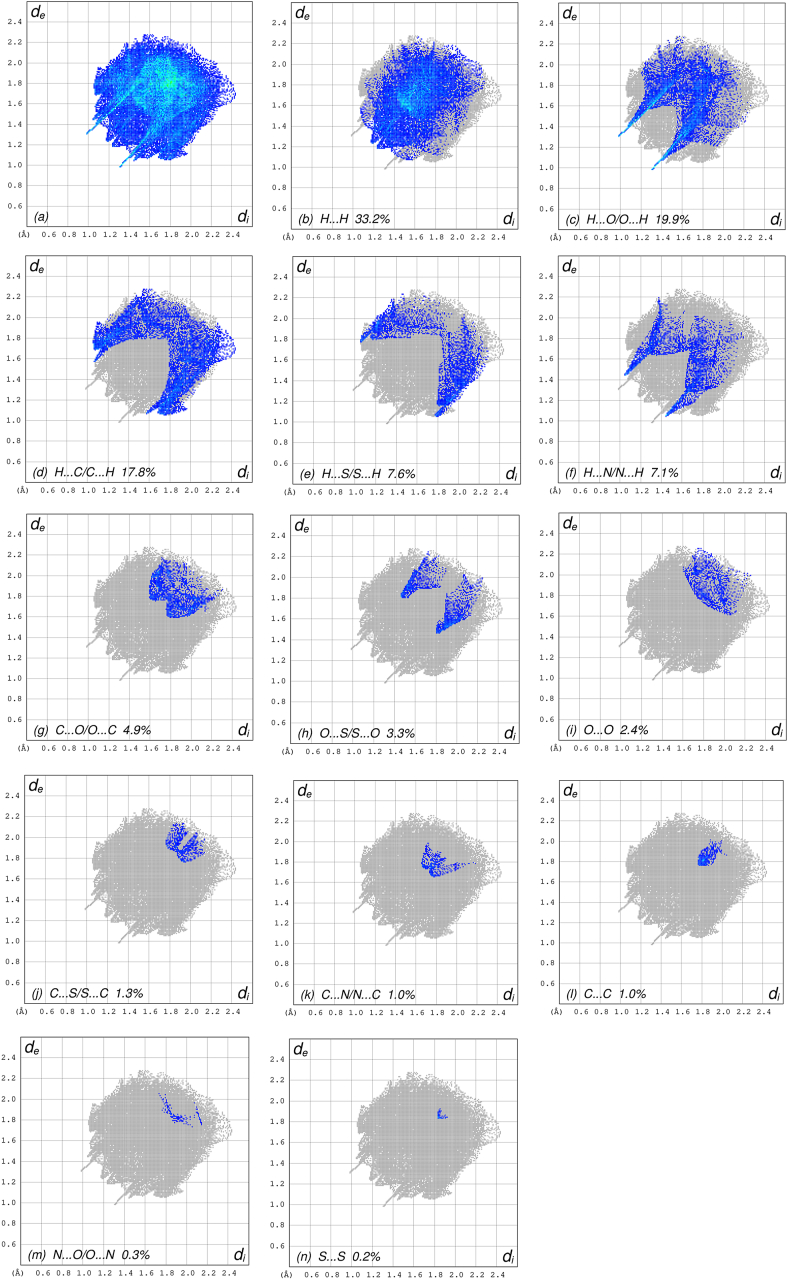
The full two-dimensional fingerprint plots for the title compound, showing (a) all interactions, and delineated into (b) H⋯H, (c) H⋯O/O⋯H, (d) H⋯C/C⋯H, (e) H⋯S/S⋯ H, (f) H⋯N/N⋯H, (g) C⋯O/O⋯C, (h) O⋯S/S⋯O, (i) O⋯O, (j) C⋯S/S⋯ C, (k) C⋯N/N⋯C, (l) C⋯C, (m) N⋯O/O⋯N and (n) S⋯S interactions. The *d*_i_ and *d*_e_ values are the closest internal and external distances (in Å) from given points on the Hirshfeld surface contacts.

In crystal structure [10], intermolecular interactions of the type C–H⋯O=C hydrogen bonds link the molecules into infinite chains along the c-axis direction. On the other hand, the C–H···π interaction may further stabilize the structure. In order to visualize the intermolecular interactions in the crystal of the title compound, a Hirshfeld surface (HS) analysis [26, 27] was carried out by using *Crystal Explorer 17.5* [28]. In the HS plotted over *d*_norm_ (Figure 5), the white surface indicates contacts with distances equal to the sum of van der Waals radii, and the red and blue colours indicate distances shorter (in close contact) or longer (distinct contact) than the van der Waals radii, respectively [29, 30]. The bright-red spots appearing near hydrogen atom H4A indicates its’ role as the respective donor and/or acceptor; they also appear as blue and red regions corresponding to positive and negative potentials on the HS mapped over electrostatic potential [31, 32], as shown in Figure 6. The blue regions indicate positive electrostatic potential (hydrogen-bond donors), while the red regions indicate negative electrostatic potential (hydrogen-bond acceptors). The shape-index of the HS is a tool to visualize the π···π stacking by the presence of adjacent red and blue triangles; if there are no adjacent red and/or blue triangles, then there are no π···π interactions. Figure 7 clearly suggests that there are no π···π interactions between the benzo[d]oxazale rings. The overall two-dimensional fingerprint plot, Figure 8a, and those delineated into H⋯H, H⋯O/O⋯H, H⋯C/C⋯H, H⋯S/S⋯H, H⋯N/N⋯H, C⋯O/O⋯C, O⋯S/S⋯O, O⋯O, C⋯S/S⋯C, C⋯N/N⋯C, C⋯C, N⋯O/O⋯N and S⋯S [33] are illustrated in Figure 8 b-n, respectively, together with their relative contributions to the Hirshfeld surface. The most important interaction is H⋯H contributing 33.2% to the overall crystal packing, which is reflected in Figure 8b as widely scattered points of high density due to the large hydrogen content of the molecule with the tip at *d*_e_ = *d*_i_ = 1.22 Å. The pair of characteristic wings resulting in the fingerprint plots delineated into H⋯O/O⋯H, Figure 8c, contacts with 19.9% contribution to the HS arises from the H⋯O/O⋯H contacts (Table 2) and is viewed as pair of spikes with the tips at at *d*_e_ + *d*_i_ = 2.28 Å. In the presence of C–H···π interactions, the pairs of characteristic wings in the fingerprint plot delineated into H⋯C/C⋯H contacts (Table 2, Figure 8d) amount up to 17.8% contribution to the HS and have sharp and spread distributions of points with the tips at *d*_e_ + *d*_i_ = 2.64 Å and *d*_e_ + *d*_i_ = 2.75 Å, respectively. The pairs of spikes in the fingerprint plot delineated into H⋯S/S⋯H contacts (Table 2) have symmetrical sharp and spread distributions of points (7.6% contribution, Figure 8e) with the tips at *d*_e_ + *d*_i_ = 2.85 Å and *d*_e_ + *d*_i_ = 2.95 Å, respectively. The pair of characteristic wings in the fingerprint plot delineated into H⋯N/N⋯H contacts (Table 2, Figure 8f, 7.1% contribution) have distributions of points with the tips at *d*_e_ + *d*_i_ = 2.48 Å. The pairs of spikes in the fingerprint plots delineated into C⋯O/O⋯C (Figure 8g, 4.9% contribution) and O⋯S/S⋯O (Figure 8h, 3.3% contribution) contacts (Table 2) have symmetrical distributions of points with the tips at *d*_e_ + *d*_i_ = 3.37 Å and *d*_e_ + *d*_i_ = 3.27 Å, respectively. The importance of C–H⋯S interactions has been recently highlighted for organosulfur compounds [34, 35] and metal complexes [36] species. The O⋯O contacts (Figure 8i, 2.4% contribution to the HS) have distributions of points without any tips with a central point at *d*_e_ = *d*_i_ = 1.77 Å. The pairs of spikes in the fingerprint plots delineated into C⋯S/S⋯C (Figure 8j, 1.3% contribution) and C⋯N/N⋯C (Figure 8k, 1.0% contribution) contacts have symmetrical distributions of points with the tips at *d*_e_ + *d*_i_ = 3.69 Å and *d*_e_ + *d*_i_ = 3.42 Å, respectively. The C⋯C contacts (1.0%, Figure 8l) have an arrow-shaped distribution of points with the tip at *d*_e_ = *d*_i_ = 1.77 Å. Finally, the N⋯O/O⋯N (0.3%, Figure 8m) and S⋯S (0.2%, Figure 8n) contacts with highly small contributions to the HS have scattered points of very low density.

**Table 2:** Selected interatomic distances (Å).

S1⋯O2^i^	3.294 (4)	O3⋯H10C	2.66
S1⋯O3	2.781 (4)	N1⋯H2A^v^	2.64
S1⋯H8A^ii^	2.94	C2⋯H10A^vi^	2.75
O2⋯H8B^iii^	2.58	C4⋯H10C^ii^	2.88
O3⋯H4A^iv^	2.43	C5⋯H10C^ii^	2.88
O3⋯H10A	2.62	C7⋯H8A^ii^	2.91

Symmetry codes: (i) *x*+1, *y*, *z*; (ii) −*x*+1, −*y*+2, −*z*+1; (iii) −*x*, −*y*+1, −*z*+1; (iv) *x*, *y*, *z*+1; (v) *x*−1, *y*, *z*; (vi) −*x*+1, −*y*+1, −*z*+1.

**Figure 9:**
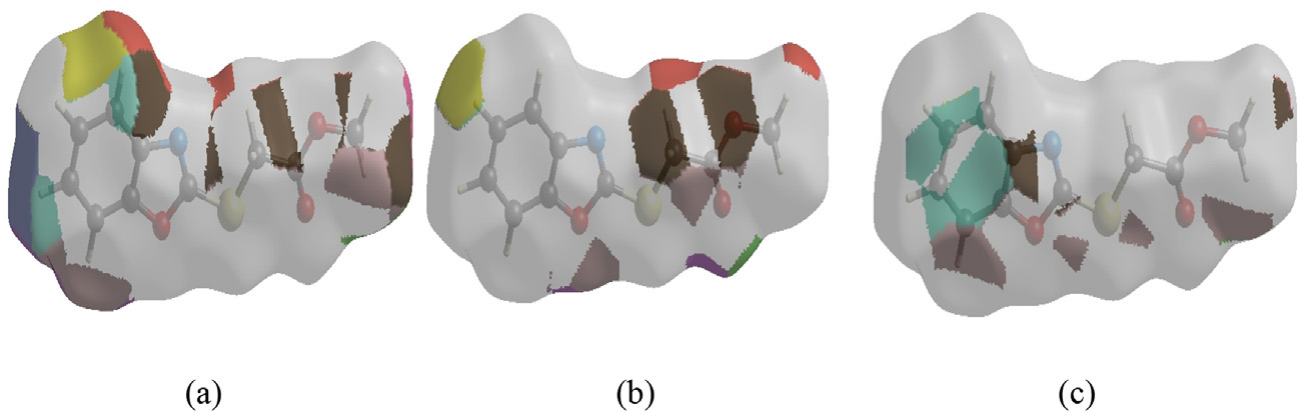
The Hirshfeld surface representations with the function *d*_norm_ plotted onto the surface for (a) H⋯H, (b) H⋯O/O⋯H, (c) H⋯C/C⋯H interactions.

The Hirshfeld surface representations with the function *d*_norm_ plotted onto the surface are shown for the H⋯H, H⋯O/O⋯H and H⋯C/C⋯H interactions in Figure 9 panels a-c, respectively. Both, the benzoxazole and methoxycarbonyl groups offer regions for strong interactions, mostly promoted by electrostatic negative potential on the oxygen atoms. Similar results were reported recently for related carboxylic species [37].

The Hirshfeld surface analysis confirms the importance of H-atom contacts in establishing the packing. The large number of H⋯H, H⋯O/O⋯H and H⋯C/C⋯H interactions suggest that van der Waals interactions and hydrogen bonding play the major roles in the crystal packing [38, 39].

### 3.4 Interaction energy calculations

In view of the relevance of the intermolecular C–H⋯O=C hydrogen bonds, the interaction energies are calculated using CE–B3LYP/6–31G(d,p) energy model available in Crystal Explorer 17.5 [28], where a cluster of molecules is generated by applying crystallographic symmetry operations with respect to a selected central molecule within the radius of 3.8 Å by default [40]. The total intermolecular energy (E_tot_) is the sum of electrostatic (E_ele_), polarization (E_pol_), dispersion (E_dis_) and exchange-repulsion (E_rep_) energies [41] with scale factors of 1.057, 0.740, 0.871 and 0.618, respectively. Hydrogen-bonding interaction energies (in kJ mol^−1^) are -17.6 (E_ele_), -3.1 (E_pol_), -62.3 (E_dis_), 49.0 (E_rep_) and -44.8 (E_tot_) for C4–H4A···O3 = O, values that are in the expected range when comparing with reported interaction energy for related compounds [42, 43].

## 4 Conclusion

The 2-(methoxycarbonylmethylthio)benzo[*d*]oxazole compound, a key intermediate towards diverse *S*-substituted heterocycles, can selectively be prepared by acylation of benzo[*d*]oxazole-2-thiol by using strong base and polar solvent. Conformational search conducted by quantum chemical calculations results indicates the planar form with the methyl thioacetate group oriented toward the C=N bond in the benzoxazole ring (synperiplanar conformation of the C7 = N1 double bond and C8–S1 single bond) as the most stable conformer, in perfect agreement with the experimental structure in the crystal phase [10]. The planarity supports an extended electronic π delocalization over the 2-thio oxazole ring and methoxycarbonyl group. The most stable conformation is stabilized by anomeric effect via the lpσ(S1)→ σ∗(C7 = N1) hyperconjugative interaction. Furthermore, a 1,4-S···O chalcogen···chalcogen intramolecular interaction is observed, characterized by short non-bonded distances of ca. 3.294 (4) Å in the crystal [10].

The Hirshfeld surface analysis of the crystal structure indicates that the most important contributions for the crystal packing are from H⋯H (33.2%), H⋯O/O⋯H (19.9%) and H⋯C/C⋯H (17.8%) interactions. Hydrogen bonding and van der Waals interactions are the dominant interactions in the crystal packing. Computational chemistry indicates that in the crystal, the C–H⋯O hydrogen-bond energy is 44.8 kJ mol^−1^. Thus, this analysis explains the occurrence of intermolecular C–H⋯O hydrogen bonds and C–H···π interactions observed in the crystal structure [10].

## Declarations

### Author contribution statement

Aamer Saeed: Conceived and designed the experiments; Performed the experiments; Analyzed and interpreted the data; Contributed reagents, materials, analysis tools or data; Wrote the paper.

Ghulam Shabir: Performed the experiments; Analyzed and interpreted the data; Wrote the paper.

Tuncer Hökelek: Conceived and designed the experiments; Performed the experiments; Analyzed and interpreted the data; Wrote the paper.

Ülrich Flörke: Performed the experiments; Analyzed and interpreted the data; Contributed reagents, materials, analysis tools or data; Wrote the paper.

Mauricio F. Erben: Conceived and designed the experiments; Performed the experiments; Analyzed and interpreted the data; Contributed reagents, materials, analysis tools or data; Wrote the paper.

### Funding statement

This work was supported by the 10.13039/501100003074ANPCyT (PICT-2019-2578) and Facultad de Ciencias Exactas, 10.13039/501100003947Universidad Nacional de La Plata for financial support (Project 11/X794).

### Data availability statement

Data associated with this study has been deposited at the Cambridge Crystallographic Data Centre.

### Declaration of interests statement

The authors declare no conflict of interest.

### Additional information

No additional information is available for this paper.
